# Panton–Valentine leucocidin expression by *Staphylococcus aureus* exposed to common antibiotics

**DOI:** 10.1016/j.jinf.2015.05.008

**Published:** 2015-09

**Authors:** Claire E. Turner, Shiranee Sriskandan

**Affiliations:** Infectious Diseases & Immunity, Imperial College London, London, United Kingdom

**Keywords:** Methicillin sensitive *Staphylococcus aureus*, Panton–Valentine leucocidin, Leucocidins, Abscess model, β-lactams, Protein synthesis inhibitors, Flucloxacillin, Clindamycin, Linzeolid

## Abstract

**Objectives:**

We set out to investigate the impact of common antibiotics on Panton–Valentine Leucocidin (PVL) expression by methicillin-sensitive *Staphylococcus aureus* (MSSA). PVL expression by methicillin-resistant *S. aureus* (MRSA) is reportedly enhanced by β-lactams, but inhibited by protein-synthesis inhibitors, a fact that has influenced management of infections associated with PVL. Although PVL is more frequently associated with MSSA than MRSA in the UK, the effect of antibiotics on PVL expression by MSSA has not been fully addressed.

**Methods:**

MSSA was cultured *in vitro* with varying concentrations of flucloxacillin, clindamycin or linezolid and PVL expression measured by qRT-PCR and Western blotting. A murine MSSA abscess model was developed to measure leucocidin expression *in vivo* following antibiotic treatment.

**Results:**

9% (27/314) of MSSA isolates from patients with uncomplicated community skin/soft tissue infections were positive for PVL genes (*lukFS-PV*). PVL expression by MSSA in broth was unaffected by varying concentrations of flucloxacillin, clindamycin or linezolid. In a murine abscess model, treatment with flucloxacillin did, however, enhance *in vivo* MSSA *lukF-PV* transcription and this was sustained even when flucloxacillin was combined with clindamycin, or clindamycin plus linezolid. Notwithstanding increased leucocidin transcription, functional leucotoxin activity was not enhanced. Treatment with flucloxacillin plus clindamycin significantly decreased leucotoxin activity, but the addition of a second protein synthesis inhibitor, linezolid, did not confer benefit.

**Conclusions:**

Our results suggest flucloxacillin in combination with a single protein-synthesis inhibitor such as clindamycin would give the best treatment outcome.

## Highlights

• 9% of MSSA isolates from uncomplicated community SST infection were PVL positive.
• MSSA PVL expression *in vitro* was not altered by β-lactam (flucloxacillin) exposure.
• Transcription of PVL *in vivo* was enhanced following flucloxacillin treatment.
• Increased PVL transcription did not lead to enhanced abscess leucotoxin activity.
• Flucloxacillin combined with clindamycin reduced leucotoxin activity *in vivo*.

## Introduction

*Staphylococcus aureus* is a globally important human pathogen that can cause a wide spectrum of diseases attributable to the range of virulence factors it is able to express. Factors that interfere with the host innate immune response are of critical importance to the success of the pathogen.

Panton–Valentine Leucocidin (PVL) is one of four pore forming bi-component toxins that may be expressed by *S. aureus* strains. The other three are gamma-haemolysin (HlgABC), LukFS (also known as LukAB or LukGH) and LukDE. The two co-transcribed components of PVL, LukS-PV and LukF-PV, when combined can lyse human cells expressing C5a receptors, including neutrophils.^1^ Strains carrying PVL typically cause suppurative skin and soft tissue infections and severe necrotising pneumonia.^2^ In north America and spreading globally, PVL has been mainly associated with strains of community acquired methicillin resistant *S. aureus* (CA-MRSA)^3,4^ but UK based studies suggest a more common association with community acquired methicillin sensitive *S. aureus* (MSSA) strains.^5^

While there is a broad literature that addresses production of PVL by CA-MRSA, reports investigating production by clinical MSSA strains are limited. Previous studies have shown that β-lactam antibiotics at sub-inhibitory concentrations can enhance MRSA transcription and expression of PVL and other toxins *in vitro*,^6–9^ although the clinical relevance of these studies to MSSA is complicated by the fact that β-lactams have no antimicrobial activity against MRSA. Antimicrobial agents targeting protein synthesis, however, have been shown to effectively reduce transcription and/or expression of PVL *in vitro*.^6,10,11^ Guidelines for treating suspected PVL MRSA infections have been influenced by these *in vitro* studies.^12^ Due to the potential effect of β-lactams on toxin expression, caution is advised with regard to use in cases of PVL-associated *S. aureus* infection and the adjuvant use of one or more protein synthesis inhibitors has been recommended.^12^ Although β-lactams are still the treatment of choice for MSSA, the effect of β-lactams and protein synthesis inhibitors on the expression of toxins in clinical MSSA has not yet been fully explored.

In this work we aimed to explore the effect of the commonly used β-lactam flucloxacillin and two protein synthesis inhibitors, clindamycin and linezolid, on MSSA expression of PVL and other leucocidins. *In vitro* exposure to each antibiotic at varying concentrations, including sub-inhibitory, did not yield a significant change in either transcription of *lukF-PV* or LukF-PV protein expression. However, *in vitro* exposure to antibiotics does not adequately reflect clinical exposure to antibiotics during infection. To this end we developed a murine abscess model to measure toxin transcription *in vivo*. Although PVL has no effect on murine neutrophils and cannot be used to model disease outcomes related to PVL, it can be used to measure *in vivo* expression of toxins and other *S. aureus* factors. In contrast to *in vitro* findings, we detected a higher level of *lukF-PV* transcript in mice treated with flucloxacillin compared to no antibiotic treatment. Surprisingly, addition of clindamycin or clindamycin plus linezolid enhanced *lukF-PV* transcript to an even greater level. Overall leucotoxin activity present in the abscess following antibiotic treatment was not affected by increased leucotoxin transcript and was in fact significantly decreased when flucloxacillin was combined with clindamycin.

## Materials and methods

### Bacterial strains

MSSA isolates (n = 314), including strains HSS03 and HSS156 used throughout this study, represented isolates from uncomplicated community SSTI collected over a one year period (2009–2010) by a single diagnostic laboratory at Hammersmith Hospital NHS Trust, London, UK (now ICHT). MSSA strains were cultured on Columbia blood agar (Oxoid) or in CCY media at 37 °C shaking at 200 rpm. Minimal inhibitory concentrations (MICs) for one MSSA PVL-positive strain collected (HSS156) were determined, in culture using a standard microdilution method,^13^ to be 0.125 mg/L for flucloxacillin, 0.25 mg/L for clindamycin and 2 mg/L for linezolid.

### DNA extraction

Bacteria were pelleted from overnight culture and resuspended in 100 μl of lysis buffer (100 mM NaCl, 10 mM Tris–HCl pH8, 1 mM EDTA, 1% Triton-X-100) with 2 μl of 1 mg/ml lysostaphin and incubated for 37 °C for 15 min before boiling for 10 min. Samples were centrifuged 13,000 × *g* for 2 min and the DNA-containing supernatant was further purified using an equal volume of chloroform. DNA was precipitated with isopropanol and resuspended in ddH_2_O. PCR using Luk-PV primers (Table 1) was performed to test for the presence of the *lukFS-PV* genes. Separate PCR reactions were also performed using positive control housekeeping 16s primers (Table 1) to control for DNA quality.

### *In vitro* exposure to antibiotics

Overnight cultures of *S. aureus* were diluted 1 in 10 then cultured for 24 h in CCY media. Where antibiotics were used, bacteria were cultured for 3 h before ¼ MIC, ½ MIC, MIC or 5x MIC of required antibiotic was added. Every hour samples were taken, centrifuged at 2000 × *g* for 10 min and culture supernatant 0.2 μM filtered. RNA was extracted from bacterial cell pellets using a hot-phenol method as previously described.^14^ Filtered supernatant was concentrated 50 fold using Amicon ultra 10 kDa MWCO spin columns (Milipore) for Western blotting.

### Recombinant LukF-PV protein

LukF-PV was amplified using LukF-recombinant primers (Table 1) and cloned into pQE-30 UA vector (Qiagen). Recombinant protein expression was induced according to the manufacturer's instructions and recombinant His-tagged LukF-PV was purified using a His-bind purification kit (Novagen).

### Western blotting

Proteins were separated by 10% Bis-Tris sodium dodecyl sulphate-polyacrylamide gel electrophoresis (SDS-PAGE) with MES buffer (Invitrogen) and transferred to nitrocellulose membrane (GE-Healthcare). Membranes were probed using rabbit polyclonal anti-LukF-PV serum raised to the C-terminal pentapeptide of LukF-PV (KNPMS) (Sigma-Genosys), the sequence of which is unique to LukF-PV. C-terminal pentapeptides have been described previously to generate specific antibodies.^15^ Blots were developed using the ECL system (GE Healthcare). LukF-PV present in culture supernatant was quantified by densitometry (ImageJ) in comparison to a standard curve of known concentrations of recombinant LukF-PV that were run alongside supernatant samples.

### Quantitative real time PCR

Bacterial RNA was treated with Turbo DNAse (Ambion) and 1 μg was converted to cDNA using Transcriptor reverse transcriptase (Roche) and random hexamer primers (Sigma). Quantitative real time PCR was performed using specific quantitative real time PCR primers for *lukF-PV*, *hlgA*, *lukF* and the house-keeping gene *rrsA* (Table 1) and SYBR green Jumpstart Taq ready mix (Sigma). Copies of target transcript were measured against a standard curve of a plasmid (pCR2.1, Invitrogen) containing the target genes *lukF-PV*, *hlgA*, *lukF* and *rrsA* of known copy number, amplified alongside bacterial cDNA. Copies of target gene transcript were then normalised to copies of the house-keeping gene *rrsA*.

### *In vivo* exposure to antibiotics

Female Balb/c mice were infected subcutaneously in the right flank with ∼2 × 10^8^ colony forming units *S. aureus* strain HSS156 in a 100 μl volume. Abscesses were allowed to form for 48 h. Mice were then treated intraperitoneally with 150 μl of either, 12.5 mg/kg flucloxacillin, 12.5 mg/kg flucloxacillin with 10 mg/kg clindamycin, 12.5 mg/kg flucloxacillin with 10 mg/kg clindamycin and 10 mg/kg linezolid, or phosphate buffered saline (PBS) as a control. To follow standard clinical treatment of infection, the flucloxacillin dose was repeated after 6 h hence, over 8 h of study, mice were treated twice with flucloxacillin. After 4 or 8 h of treatment mice were culled and width, height and depth of abscesses were measured using callipers to calculate abscess volume. Pus was then extracted from abscesses and a sample diluted and plated for bacterial enumeration which was then calculated as total bacterial CFU per entire abscess volume. Remaining pus was diluted in TE buffer and RNA was extracted using a hot-phenol method previously described^14^ or reserved for measuring lysis activity. RNA was cleaned using RNeasy MinElute cleanup kit (Qiagen) then 1 μg converted into cDNA as described above.

### Leucocidin activity

Total leucotoxin activity within pus from murine abscesses was tested using PLB-985 cells (HL-60-like myeloid leukaemia cell line). PLB-985 cells were cultured in RPMI+ with 10% FCS in 5% CO_2_ and differentiated to a neutrophil-like phenotype with 1.25% DMSO.^16^ Cells were washed and resuspended to 1 × 10^7^/ml in RMPI with HEPES. For each pus sample, two aliquots of 245 μl RMPI + HEPES with 5 μl of pus were made; one was heated to 100 °C for 10 min to inactivate the heat-labile staphylococcal leucotoxins (including PVL) and control for background leucotoxin activity. 100 μl heated or non-heated pus aliquot was added to 100 μl PLB-985 cells in duplicate in round bottom 96 well plates and incubated for 3hrs at 37 °C, 5% CO_2_. Samples were then centrifuged at 700 **g** for 5 min and 100 μl supernatant was transferred to flat bottom 96 well plates. LDH levels were then measured in the supernatant using a cytotoxicity detection kit (Roche) with an LDH standard curve of known LDH concentrations to quantify.

## Results

### Prevalence and expression of PVL toxins among MSSA

Between 2009 and 2010 MSSA isolates were collected from patients with uncomplicated skin infections. Of the 314 strains tested, 9% were positive for PVL.

**Figure 1:**
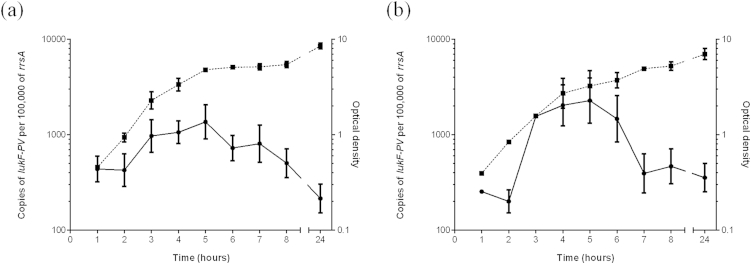
**Copies of LukF-PV transcript during exponential growth**. Two clinical MSSA PVL + strains (a) HSS156 and (b) HSS03 were cultured through exponential growth (Optical density; dotted line) and copies of *lukF-PV* transcript were measured by quantitative real time PCR at each hour, normalized to copies per 100,000 copies of the housekeeping gene transcript *rrsA* (Solid line). Copies of *lukF-PV* transcript increased during exponential growth before peaking at mid-late exponential phase. Similar results were observed in both strains although strain HSS156 (a) expressed fewer copies of transcript than strain HSS03 (b). Data represent mean (±standard deviation) of five (HSS156) or two (HSS03) individual experiments.

**Figure 2:**
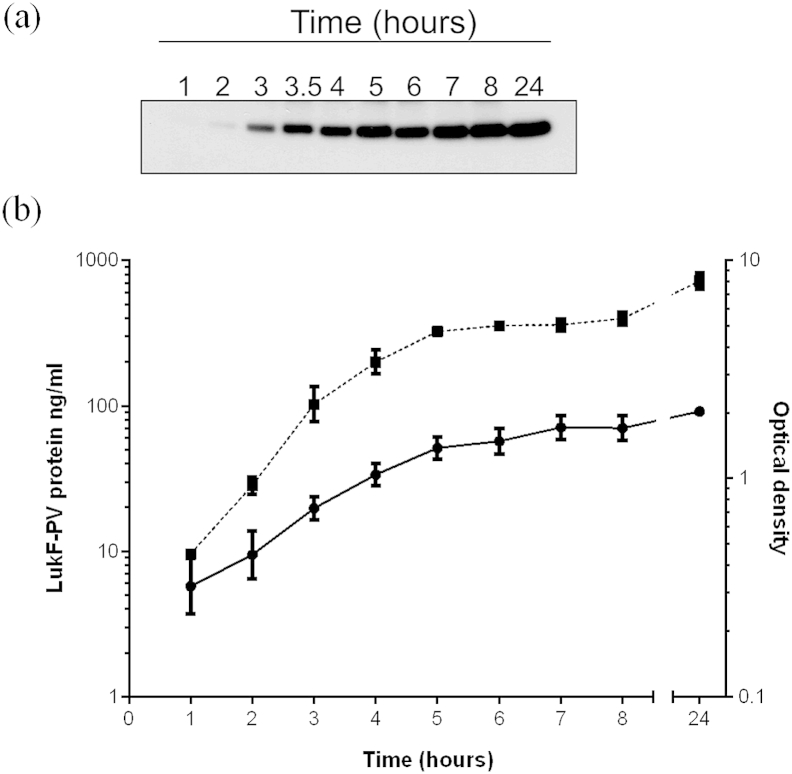
**LukF-PV protein accumulates over exponential growth**. (a) The amount of LukF-PV protein present in the culture supernatant of strain HSS156 at each time point during exponential growth was measured by Western blot. Recombinant LukF-PV protein of known concentrations was measured alongside in order to quantify the amount of LukF-PV by densitometry. (b) The amount of LukF-PV protein (ng/ml) increases in the culture supernatant (solid line) during exponential growth, measured by optical density (dotted line, A_600nm_). Data represent mean (±standard deviation) of five individual experiments.

To determine the pattern of expression of LukF-PV during culture, two clinical MSSA PVL positive strains (HSS03 and HSS156) from the community uncomplicated SSTI collection were cultured through exponential growth. For both strains, levels of *lukF-PV* transcript increased during the exponential growth phase and peaked at mid-late exponential phase (Fig. 1). The amount of LukF-PV protein present in the culture supernatant of strain HSS156 continued to accumulate during exponential growth and did not diminish after overnight culture (Fig. 2).

### *In vitro* effect of antibiotics on PVL expression

In order to test the effect of antibiotics on the expression of PVL *in vitro*, MSSA strain HSS156 was cultured to mid-exponential phase (3 h) when expression of LukF-PV was increasing. Flucloxacillin, clindamycin or linezolid were then added at different concentrations; ¼MIC, ½ MIC, MIC and 5 x MIC.

**Figure 3:**
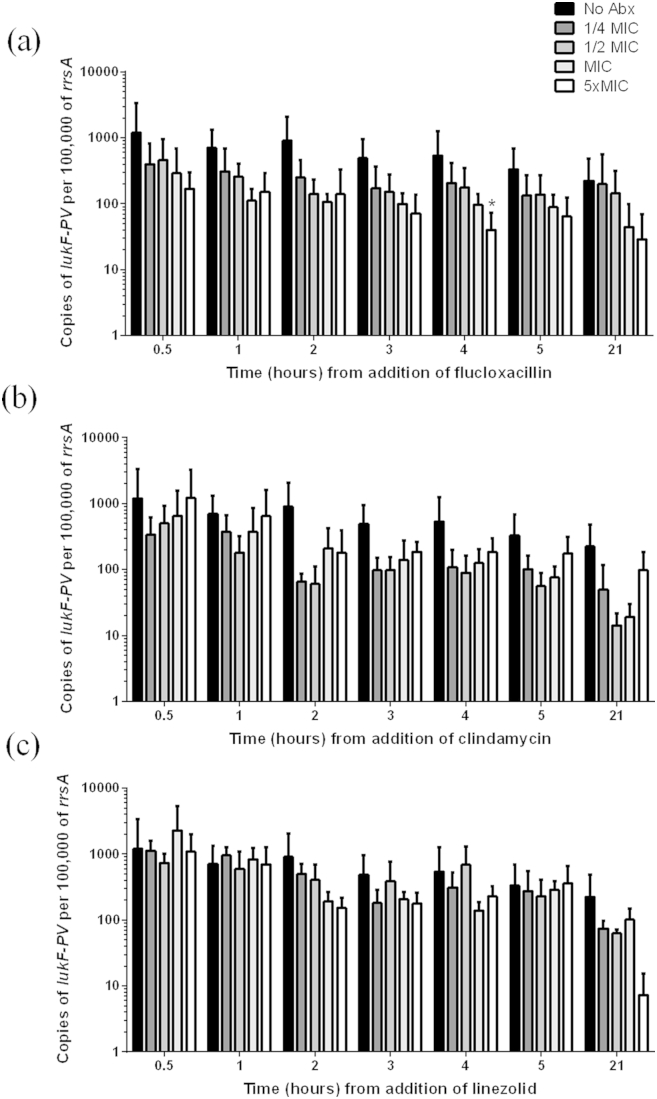
**The effect of antibiotic exposure on *lukF-PV* transcription *in vitro***. Flucloxacillin (a), clindamycin (b) or linezolid (c) was added to a culture of HSS156 in mid-exponential phase of growth (3 h) at four concentrations; ¼ of the minimal inhibitory concentration (MIC, dark grey bars), ½ MIC (lighter grey bars), MIC (lightest grey bars) and 5x MIC (white bars). Copies of *lukF-PV* transcript were measured by quantitative real time PCR and normalized to copies per 100,000 of the house-keeping gene *rrsA* transcript. Data represent the mean (+standard deviation) of 3–5 independent experiments. *p ≤ 0.05 compared to no antibiotics (No Abx, black bars) at the same time point (Kruskal–Wallis).

The addition of flucloxacillin, clindamycin or linezolid resulted in either equal or reduced levels of *lukF*-*PV* transcript compared to the no antibiotic control at all time points and all concentrations of antibiotic (Fig. 3). Following two hours exposure to either flucloxacillin or clindamycin at all concentrations there was some reduction in *lukF-PV* transcript, although only flucloxacillin 5xMIC at 4hrs resulted in a significant reduction. Linezolid had little impact on *lukF-PV* transcript levels with only some reduction after 21 h of exposure suggesting a delayed effect of linezolid *in vitro*.

**Figure 4:**
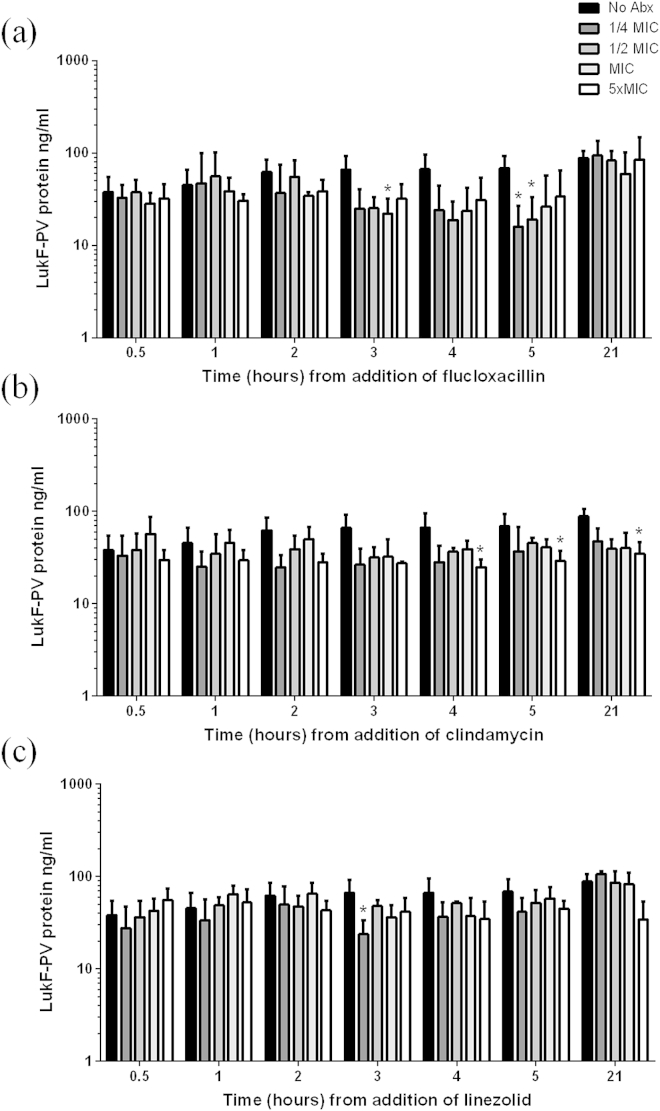
**The effect of antibiotics on LukF-PV protein *in vitro***. Flucloxacillin (a), clindamycin (b) or linezolid (c) was added to a culture of HSS156 in mid-exponential phase of growth (3 h) at four concentrations; ¼ of the minimal inhibitory concentration (MIC, dark grey bars), ½ MIC (lighter grey bars), MIC (lightest grey bars) and 5x MIC (white bars). LukF-PV protein concentration was measured by Western blotting and densitometry in comparison to a standard curve of recombinant LukF-PV. Data represent the mean (+standard deviation) of 3–5 independent experiments. *p ≤ 0.05 compared to no antibiotics (No Abx, black bars) at the same time point (Kruskal–Wallis).

The accumulation of LukF-PV protein in the culture supernatant was also measured (Fig. 4). A reduction in LukF-PV protein was observed following 3 h exposure to either flucloxacillin or clindamycin although this failed to reach significance except at some time points (flucloxacillin; MIC at 3 h, ½MIC and ¼MIC at 5 h, clindamycin; 5 x MIC at 4, 5 and 21 h). Linezolid appeared to have little effect on LukF-PV protein levels despite the mode of activity of this antibiotic.

Examination of the culture media after 21 h of antibiotic exposure revealed that the addition of flucloxacillin increased the total protein content in culture media, possibly due to β-lactam mediated bacterial cell death releasing intracellular proteins (Supplementary Figure S1). Thus, to take into account the effect on bacterial growth, we measured total LukF-PV protein present in the culture supernatant without adjusting for the optical density or number of bacteria as this measurement includes the active expression and release of LukF-PV as well as intracellular LukF-PV released during cell death.

### *In vivo* effect of antibiotics on leucocidins

**Figure 5:**
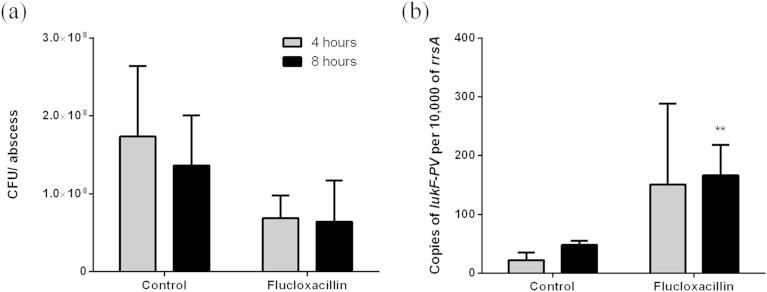
**Flucloxacillin enhances LukF-PV expression *in vivo***. Mice were infected subcutaneously leading to abscess formation. Treatment with flucloxacillin only slightly reduced total abscess bacterial burden (a) compared to control (phosphate buffered saline, PBS) after 4 h (grey bars) and 8 h (black bars) following treatment. Copies of *lukF-PV* transcript within the abscess however were increased following treatment with flucloxacillin to a significant level by 8 h of treatment (b). Data represent mean (+standard deviation), n = 6 mice per group and time point. **p < 0.01 Kruskal–Wallis compared to control at 4 h.

The concern over the effect of sub-inhibitory concentrations of antibiotics on toxin production is founded in part on the possibility that, during severe clinical disease, the appropriate antibiotic dose may not reach the site of infection. To test how a standard dose of antibiotic would affect MSSA toxin expression during disease we developed a murine abscess model of MSSA that can be used to measure bacterial toxin transcription *in vivo*. Mice were infected subcutaneously with MSSA strain HSS156 and over 48 h a well circumscribed pus-filled abscess formed. At this time point mice were then treated with flucloxacillin, or phosphate buffered saline (PBS) as a control. Four or eight hours later mice were culled and abscesses were drained for bacterial enumeration and RNA extraction. There was only a marginal, non-significant effect of flucloxacillin treatment on total abscess bacterial load (Fig. 5a) possibly related to the short time frame of the experiment. In contrast to the *in vitro* findings however, there was an increase in the level of *lukF-PV* transcript in the abscesses of mice treated with flucloxacillin compared to control PBS treated mice by four hours, reaching a significant 3.5 fold increase by 8 h (Fig. 5b).

**Figure 6:**
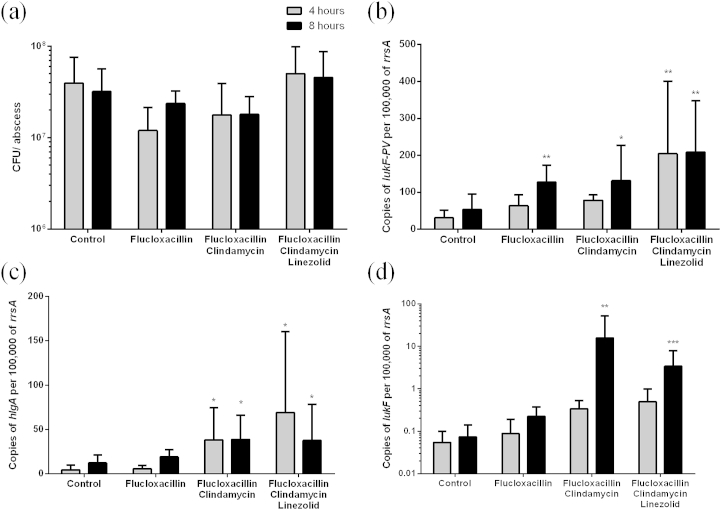
**Antibiotic treatment affects transcription of leucotoxins**. Mice were treated with either flucloxacillin or flucloxacillin in combination with clindamycin or clindamycin with linezolid. (a) All antibiotic combinations had little effect on the bacterial load within the abscess in the short time period of 4 h (grey bars) and 8 h (black bars). Copies of *lukF*-*PV* transcript (b), *hlgA* transcript (c) and *lukF* transcript (d) were measured within the abscess after 4 h treatment or 8 h treatment. Data represent mean (+standard deviation), n = 6 mice per group and time point. *p ≤ 0.05 **p ≤ 0.01 ***p ≤ 0.001 Kruskal–Wallis compared to control at 4 h.

UK guidelines for treating severe PVL-associated *S. aureus*, such as necrotising pneumonia, recommend the avoidance of β-lactams and inclusion of clindamycin and linezolid to reduce the level of toxins produced by the bacteria.^12^ To test the effect of antibiotic combination treatment on PVL toxin production, the *in vivo* experiment was repeated but this time groups of mice were treated with flucloxacillin, flucloxacillin with clindamycin, flucloxacillin with clindamycin and linezolid combined or PBS as a control. There was still little effect of antibiotic combinations on the bacterial burden within abscesses (Fig. 6a). The level of *lukF-PV* transcript was again significantly enhanced by flucloxacillin treatment by 8 h but this increase did not diminish when given in combination with clindamycin (Fig. 6b). Indeed transcript levels of *lukF-PV* were further enhanced when flucloxacillin was combined with clindamycin and linezolid. To determine if this was specifically an effect on *lukF-PV* we also measured the level of transcripts for gamma haemolysin (*hlgA*) (Fig. 6c) and *lukF*, another bi-component leucocidin (Fig. 6d). Although flucloxacillin alone did not affect the transcription of *hlgA* and *lukF*, in combination with clindamycin or clindamycin and linezolid the level of transcript of both toxin components were enhanced.

**Figure 7:**
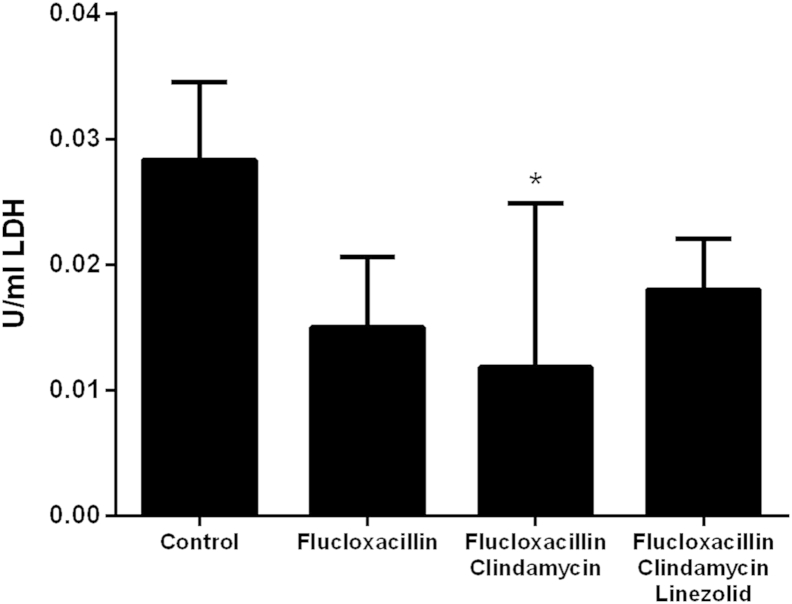
**Residual lytic activity present in pus is reduced by antibiotic treatment**. Pus was extracted from the abscesses of mice treated with either flucloxacillin or flucloxacillin in combination with clindamycin or clindamycin with linezolid for 8 h and tested for lytic activity against the cell line PLB-985. Cell lysis was measured by LDH release (units of LDH per ml, U/ml). Data represent mean (+standard deviation), n = 6 mice per group. *p ≤ 0.05 Kruskal–Wallis compared to control (PBS treated mice).

The increase in transcript copy number may not, however, necessarily translate to an increase in toxin production. Unfortunately we were unable to detect LukF-PV protein in pus obtained from abscess by Western blot, therefore, as a surrogate for protein expression, we quantified total leucotoxin activity present in each abscess by exposing the human neutrophil-like cell line PLB-985 to pus from infected mice and measuring LDH release. Treatment with all combinations of antibiotic reduced overall leucotoxin activity present in pus (Fig. 7) however only flucloxacillin with clindamycin reduced this to a significant level compared to the control PBS treated mice.

## Discussion

Previous *in vitro* studies focussing on CA-MRSA strains have shown that sub-inhibitory concentrations of β-lactam antibiotics can enhance expression of PVL and other toxins, whereas protein synthesis inhibitors reduce expression of toxins.^6–11^ Guidelines for treating suspected PVL-MRSA infection have been influenced by these studies yet there have been limited reports regarding the effect on clinical MSSA.

In the UK the dominant source of PVL is MSSA, particularly associated with SSTI.^5^ We tested community SSTI-associated MSSA strains and found 9% were positive for PVL toxin genes. The level of PVL protein expression by one clinical MSSA strain was ∼100 ng/ml, similar to that of previously tested CA-MRSA (50–350 ng/ml).^17^

Following exposure to antibiotics *in vitro*, we did not find a significant effect of the β-lactam flucloxacillin on PVL expression in a clinical MSSA strain, at sub-inhibitory concentrations or otherwise, although in some instances transcription appeared to be marginally reduced. This is in contrast to previous studies indicating enhanced transcription of PVL following exposure of CA-MRSA to β-lactam antibiotics.^7–10^ This suggests a difference in response to β-lactams between MSSA and CA-MRSA but could also be due to different methods and time points tested. We also demonstrated a similar effect on MSSA PVL transcription following exposure to clindamycin and linezolid. The effect of all three antibiotics on PVL protein expression was also limited at all concentrations and time points, remaining equal or marginally reduced compared to the no antibiotic control. This was unexpected in the case of clindamycin and linezolid, given their mode of action but may reflect a delayed or more subtle response in liquid culture. A previous *in vitro* study using simulated clinical doses found at much later time points (48 and 72 h), linezolid actually increased PVL transcription by CA-MRSA although decreased PVL protein expression.^11^

Exposure to and subsequent effects of systemic antibiotics *in vivo* are likely to be quite different to the effects of antibiotics in broth *in vitro*. The murine subcutaneous abscess model allowed us to effectively measure the level of toxin transcript produced by MSSA during abscess formation. Surprisingly, we did identify an increase in the level of *lukF-PV* transcript following treatment of mice with flucloxacillin compared to mice that received only PBS as a control. This is consistent with a previous study identifying an increase in PVL transcription in a murine lung infection model following treatment with imipenem.^18^ Enhancement of *lukF-PV* transcript was more pronounced when clindamycin was included in the treatment and even more so when clindamycin and linezolid were combined with flucloxacillin.

PVL was not the only toxin to be affected as an increase in transcript of gamma-haemolysin component *hlgA* and *lukF* were also observed. LukF of LukFS (not PVL) is also known as LukG from the bi-component toxin LukGH^19^ or LukB from the bi-component toxin LukAB.^20^ In our MSSA strain LukF was transcribed at a much lower level compared to LukF-PV.

The LukS component of PVL targets human C5a receptors on the surface of cells, hence the cytotoxic activity of PVL is restricted to cells expressing this receptor. Although PVL is cytotoxic towards human and rabbit neutrophils it displays no activity towards murine neutrophils.^1^ Our murine abscess model is not intended for study of the activity and downstream effects of PVL *in vivo* and we did not look at disease outcomes. Instead the model was used as an *in vivo* biological system to evaluate toxin production.

We were unable to detect LukF-PV in abscess pus by Western blot and we therefore used a surrogate test of measuring leucotoxin activity of pus towards a human neutrophil-cell line PLB-985. This test measured total leucotoxin activity and was not restricted to PVL. Flucloxacillin in combination with clindamycin significantly reduced the ability of abscess pus to lyse PLB-985 cells. When linezolid was also included in treatment, this reduction in lytic activity was lost. It has been shown previously that linezolid and clindamycin act synergistically to reduce toxin expression.^21^ Our study, although limited by a single MSSA strain, does however suggests that, at least in the presence of flucloxacillin, this may not be the case.

The method used to measure transcription in the current report measured transcript copy number in relation to an *S. aureus* housekeeping gene, thus this does not take bacterial abundance into consideration. As such, the impact of transcription may be heavily tempered by bacterial abundance and viability. Although the changes were not significant, the reductions observed in bacterial CFU or a bacteriostatic effect when using antibiotics may have been sufficient to influence total protein production. This would be consistent with our observation of the paradoxical increase in toxicity when using all three antibiotics, since this combination resulted in a non-significant increase in bacterial CFU. Additionally, protein synthesis inhibitors can cause an increase in transcription due to disrupted transcriptional regulation but their mode of action means no consequent enhancement of protein production.

Further work is required to identify if clindamycin alone is sufficient to reduce cytotoxicity or if the detrimental effect of linezolid in this study was due to an agonistic effect with clindamycin. The effect of rifampicin, which is also recommended by clinical guidelines for intracellular bacterial clearance could also be evaluated in this model,^12^ and it is unclear if the observed *in vivo* effects on MSSA would apply to CA-MRSA; this requires further study. Our findings do suggest that, for skin and soft tissue infections where MSSA-PVL is suspected that combined treatment of flucloxacillin and clindamycin would be the most effective but that the additional inclusion of linezolid for treatment of MSSA may be unnecessary.

## Conflict of interest

The authors declare no conflict of interest.

## Figures and Tables

**Table 1:** Primers used in this study.

Primer name	Primer sequence (5′-3′)
16s- forward	CGGTCCAGACTCCTACGGGAGGCAGCA
16s- reverse	GCGTGGACTACCAGGGTATCAATCC
Luk-PV1	ATCATTAGGTAAAATGTCTGGACATGATCCA
Luk-PV2	GCATCAACTGTATTGGATAGCAAAAGC
LukF- recombinant-F	GCTCAACATATCACACCTGTAAGTG
LukF-recombinant-R	TTAGCTCATAGGATTTTTTTCCTTAG
rrsA-F (qPCR)	AGCTTAGTTGCCATCATTAAGTTGG
rrsA-R (qPCR)	GTTGAGACTACAATCCGAACTG
LukF-PV F (qPCR)	AAGCTGCTGGAAACATTTATTCTGGC
LukF-PV R (qPCR)	CTGAATCTGAATTAATTGAAATGTTGTACTTAGAA
LukF F (qPCR)	ATTGGAATAGTACATTAAGATGGCCTG
LukF R (qPCR)	CTCCACGATTAATCGAAAAATCTC
hlgA F (qPCR)	GCAGTTGGTTTAATAGCCCCTTTAG
hlgA R (qPCR)	GTTATAGCTAATCGTTTGCTAGTAATGTCTTG
