# Supplementary material for: Panton–Valentine leucocidin expression by Staphylococcus aureus exposed to common antibiotics
Source: J Infect. 2015 Sep;71(3):338–46. doi: 10.1016/j.jinf.2015.05.008 (PMC4535317; doi:10.1016/j.jinf.2015.05.008)
Supplement: Supplementary file 1 [file mmc1.pdf]

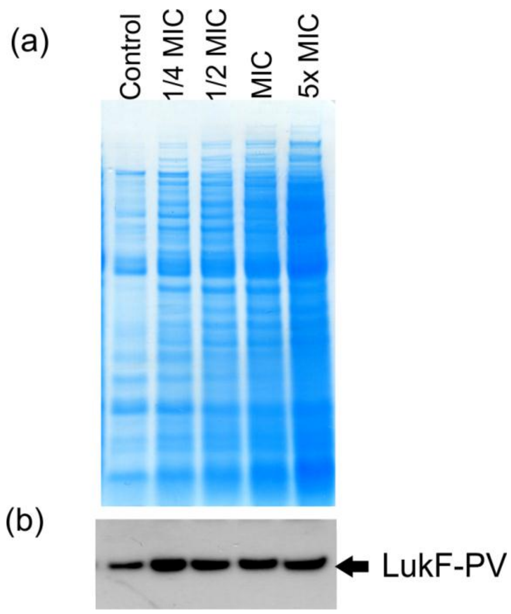

Supplementary Figure S1

Increasing flucloxacillin concentration results in increased total protein content of culture supernatant associated with an apparent increase in PVL when samples are adjusted for growth optical density. Flucloxacillin was added to a culture of HSS156 in mid-exponential phase of growth (3 hours) at four concentrations; ¼ of the minimal inhibitory concentration (MIC), ½ MIC, MIC and 5x MIC. After 21 hours of exposure to flucloxacillin, cultures were adjusted to the same optical density (A_{600nm}10) and concentrated cell-free culture supernatants were subjected to SDS-PAGE and Western blotting. (a) SDS-PAGE gel of each supernatant sample stained with Colloidal blue. (b) Western blot for LukF-PV of equivalent SDS-PAGE gel.
